# Understanding the Interactions of Happiness, Self-Rated Health, Mental Feelings, Habit of Eating Healthy and Sport/Activities: A Path Model for Abu Dhabi

**DOI:** 10.3390/nu14010055

**Published:** 2021-12-23

**Authors:** Masood A. Badri, Mugheer Alkhaili, Hamad Aldhaheri, Hamdan Alnahyan, Guang Yang, Muna Albahar, Asma Alrashdi

**Affiliations:** 1 Department of Business Administration, College of Business and Economics, United Arab Emirates University, Al Ain P.O. Box 88888, United Arab Emirates; Muna.Albahar@uaeu.ac.ae; 2 Abu Dhabi Department of Community Development, Abu Dhabi P.O. Box 15551, United Arab Emirates; Mugheer@addcd.gov.ae (M.A.); Hamad.aldhaheri@addcd.gov.ae (H.A.); Hamdan.alnahyan@addcd.gov.ae (H.A.); Guang.yang@addcd.gov.ae (G.Y.); Asma.alrashdi@addcd.gov.ae (A.A.)

**Keywords:** self-rated health, happiness, physical activities, mental health, eating healthy, path analysis, Abu Dhabi

## Abstract

Understanding the interactions between happiness, self-perception of health, healthy eating behaviors, physical activities, and psychological feelings or symptoms of mental health provides necessary inputs for social policymaking. Using data from the second cycle of the Abu Dhabi Quality of Life survey, this study examined a path analysis of Abu Dhabi residents’ nature of assimilations between these variables. The results point to the significant association between happiness and self-rated health. In addition, the results portray significant direct paths to happiness from three mental feeling variables—“feeling calm and peaceful”, “having lots of energy”, and “feeling downhearted and depressed”. The variable of “often feeling rushed or pressed for time” shows a direct path to self-rated health only. Eating healthy food is significantly associated with both happiness and self-perception of health. In addition, “often doing physical activities” positively influences happiness. The present study enhances and refines policymakers’ understanding of the considered factors on happiness and self-rated health with further elaborations of the mediating roles of specific well-being determinants. Limitations and future research directions are also discussed.

## 1. Introduction

A better understanding of the determinants of happiness amongst people in a community could provide social policymakers with standard metrics that help analyze and compare the effects of different policies [1,2,3]. In addition, measuring and tracking happiness can be especially helpful to multiple stakeholders involved in well-being and health promotion [4,5,6]. In the literature, abundant studies aim to understand the association of some personal behaviors, feelings, and habits with happiness. For example, [7] reviewed the relationships between happiness and sports and physical activities, while [8] synthesized research findings related to the impact of eating healthy food on happiness. Others, such as [9], analyzed happiness as a predictor of the mortality of the elderly adjusted for health and physical activity, while [10] discussed the role of certain mental feelings or intrinsic motivation indicators in influencing well-being.

According to the *How’s Life* report from the Organization for Economic Co-operation and Development [11,12], many researchers in their analyses of people’s happiness use data collected through quality-of-life surveys, which have been a significant data source for many well-being studies [13,14]. However, some international research usually concentrates on selected social indicators that do not include health-related indicators [6,13], whereas an ideal set of health indicators could provide useful information about physical and mental health outcomes [15,16]. Self-perception of health status is relatively well covered in research projects and regularly collected across OECD countries, using a standard answering scale. The OECD, for example, measures self-reported mental health status indicators such as individuals feeling calm and peaceful, feeling active and full of energy, feeling downhearted and depressed, and rushed for time [12]. 

The UAE has paid significant attention to the happiness index by devoting a stand-alone ministry—The Ministry of Happiness—to this area of research [17,18]. Meanwhile, in the Emirate of Abu Dhabi, a Well-being Committee is formed to report well-being and happiness-related strategies to the executive leadership of the Emirate [19]. As a result, the Abu Dhabi Quality of Life survey is conducted annually by the Abu Dhabi Department of Community Development and the Abu Dhabi Statistics Department, which primarily reflects the main components of the OECD Better Life indicators. 

This research concentrates on several main factors related to happiness: self-rated health, subjective mental feelings, physical activities/sport, and eating healthy food. The objective is to carefully design and test a path model to understand the variables’ associations when taking happiness as the outcome. 

## 2. Review of Literature

In a framework within well-being, many researchers focus on happiness as experiencing positive feelings throughout the day (or days) in contrast to general life satisfaction, which reflects a sense of purpose in life [20,21,22]. In addition, happiness has been regarded as one of the most essential and fundamental goals in life [4]. Some regard increased happiness as an essential precursor of general health [20]. Therefore, it is vital to truly understand the factors that promote and increase our happiness. In the context of the present study, the focal concept of happiness is the subjective assessment of life satisfaction [22]. This concept facilitates researchers to measure happiness related to the quality-of-life abstract domain [23,24]. In addition, some view happiness as the experience of satisfaction, and this satisfaction can come from everything around a person as we refer to the quality of life [25].

Research confirms that happiness and health share significant similarities in many well-being determinants [26,27]. Primarily, research has identified income, social connections, mental health, and physical and sport factors linked with both happiness and health [28]. Moreover, the relation between health and happiness has also been vastly studied but with inconsistent results when considering direction and magnitude [26]. *Happiness* is generally defined as a joyful state of mind that reflects an individual’s overall subjective well-being [4,22,29] and is increasingly considered an essential tool to guide public policy and measure the effectiveness of policy actions [30]. Many countries (i.e., Canada, France, and the UK) have included a national happiness index to measure national progress [22]. Internationally, the World Happiness Report 2021, based on the Gallup World Poll and a wide variety of data sources, identifies the happiness indicators of individuals in countries around the globe [31]. 

The concept of relations between happiness and health has been studied from different approaches. Most have analyzed the correlations between them. For example, some reported that Argyle (1997) happiness affects health and vice versa [32]. However, most research discussing health referred to physical and mental health [33,34]. There is a wealth of studies on the association between happiness and subjective health [29]. However, research that dealt with the association between happiness and self-rated health provided different results. Most studies that included paths (between happiness and self-rated health) established paths from self-rated health to happiness. For example, for elderlies, [35] reported an indirect path from health to happiness, but through hedonic balance [36]. In a similar study in China on elderlies, [37] concluded a path from self-rated health to happiness. 

In a study testing the relationship between happiness and self-rated health in Italy, a direct path from happiness to self-rated health was recorded [36]. However, some used the word “debate” to point to the bidirectional relationship between happiness and health [38]. The author pointed out that happier people feel healthier than unhappier people. In addition, health can influence happiness and life satisfaction.

Extensive studies have highlighted the health benefits of happiness [39]. Research findings tend to show that happiness promotes a range of lifestyle practices and patterns that affect overall health [40]. Therefore, policymakers are encouraged to enable new channels to investigate health promotions using physical activity interventions [41]. 

The benefits of physical activity on health have been well documented. For example, active people enjoy better health and are happier than inactive peers [42]. Moreover, many suggest that physical activity is correlated with significant health benefits across an individual’s life course [43,44]. For example, [45] suggested that simple regular activity has been shown to play a significant role in preventing many diseases, such as cancer, osteoporosis, cardiovascular disease, diabetes, and obesity. 

The health benefits of happiness extend to mental health. The World Health Organization (WHO) points to the belief that “a healthy mind can represent a healthy body” [46]. As a subjective state of mind, happiness is associated with psychological or mental health, and many studies elaborate on how positive emotions correlate with happiness [47]. Some explain that the concept of mental health is also subjective and argue that happiness is one of the most important reflections and aspects of positive psychology [48]. Some authors suggest that happiness is a subjective emotional outcome of many different mental feelings such as stress, anxiety, and depression and that happy people have less chance to be prone to mental disorders [49,50].

Many studies show that sports and physical activities have a significant association with happiness [51]. Some research evidence points to the association between regular participation in physical activities and positive mental-health-related outcomes such as anxiety, stress, depression, enhanced cognitive function, and academic performance [42,52,53,54]. The effects of physical activity on happiness were examined for different categories of people, including adolescents [55] and older adults [56], generally revealing positive associations. Some found that total minutes of physical exercise per week was positively related to happiness [57]. A review of research on happiness and physical activity showed that a positive direct or indirect association between happiness and physical activities is consistently found in the literature [7]. However, they could not confirm the presence of a causal relationship between physical activity and happiness. While researchers encourage more research to investigate whether physical activity might be an essential correlate of happiness, some refer to social interactions gained through physical activities that lead to one’s happiness [56,58]. In addition, most extant studies tend to focus on the association of physical activity with the negative aspects of mental health [59,60]. 

Research shows that those with a positive well-being were more likely to consume healthy food (i.e., fresh fruits and vegetables) than their less favorable counterparts [61]. The WHO characterizes healthy eating or healthy diet as involving diet, fruit, and vegetables, a less or moderate amount of fats and oil, and less salt and sugar [62]. Studies show that diets rich in fruits and vegetables have consistently been associated with a range of health benefits, including lower risks of diabetes, stroke, and heart disease [63,64,65]. Thus, there is significant evidence that encourages healthy eating and suggests that healthy food choices should be considered as a long-term investment in future well-being [66,67,68]. Some research investigated the tradeoff between eating healthier food and happiness. For example, ref [8]’s review of research reports on the nature of the relationship between healthy food and happiness provides clear evidence of a strong association between healthy eating and happiness, where such a pattern seems to be universal and only with minor variations across time, people, and regions in the world. Research also points out that the relationship between eating healthy food and our mental health might be somehow complex. Existing research shows a link between what we eat and how we feel [69], and suggests that healthy, well-balanced food could contribute to our ability to stay alert, concentrate, and pay attention [70] and an inadequate food diet could lead to more fatigue, stress, depression, impaired decision-making, and slower reaction time [64,71].

Although many studies recognized health and happiness individually and in isolation, some existing research took a more profound understanding to further investigate the direction of effect between the two outcomes: does happiness affect health or vice versa? [72,73,74]. In this study, we investigate this understanding between health and happiness using an alternative conceptual framework, which includes the interaction of other related factors, such as subjective mental feelings, often doing sports, and eating healthy food. We would investigate the role of those other factors described by many studies as fundamental in shaping such vast relationships. We would consider subjective health and happiness as distinct yet associated with each other, with the mediations of other well-being factors. Such an investigation would enrich our understanding of those other social forces (such as mental health, eating healthy, and physical activities). 

The World Happiness Report also acknowledged some Arab countries in its list, where the UAE enjoyed the top spot with a score of (6.825) and ranked 21st globally [75]. A total of 19 Arab countries were listed in the report. The UAE has been taking significant steps to promote happiness. In 2016, the UAE appointed its first-ever minister of happiness. Its main objectives were to promote and sustain happiness for all and throughout the UAE. In addition, in March 2017, the UAE launched the World Happiness Council, which had to improve the state of happiness throughout the UAE and across the world. The council focused on health, education, environment, personal happiness, happy cities, and community standards for happiness. As determinants of happiness, [76] explored the relationship between Islamic religiosity and satisfaction with a diverse range of life and health domains. They concluded that religiosity has a robust positive relationship with subjective well-being. Regarding the UAE, a happiness-related research paper addresses the factors associated with the subjective well-being of older adults in Abu Dhabi.

This research aims to contribute to the literature by focusing on these specific dimensions related to happiness, including self-rated health, healthy eating, physical activities, and mental feelings. Drawing on an extensive survey of Abu Dhabi residents examines the associations between these subjective indicators and happiness. In addition, the strength of relationships will also be examined. The results of this study could provide inputs and evidence to inform the social policy-making process in Abu Dhabi that has given priority to the improvement of the lives and well-being of Abu Dhabi residents. 

## 3. Materials and Methods

### 3.1. Survey and Data Collection

The second set of Abu Dhabi Quality of Life (QoL) survey data was considered for this research. Based on some international well-being frameworks and general social surveys, including the OECD’s Better Life, World Happiness Report, Gallup Global Well-being Survey, and European Quality of Life Surveys, the Abu Dhabi Quality of Life Survey covered a variety of dimensions and factors that are believed to affect the well-being of residents of Abu Dhabi. Those dimensions range from housing, household income, jobs, and earnings, to health, education, safety, and social connections. The survey was administered online from September 2019 to March 2020. It covered residents aged 15 or above in all regions of the Emirate of Abu Dhabi. Both the Department of Community Development (DCD) and the Statistics Center Abu Dhabi (SCAD) provided the ethical approval for this study. The study sample included residents across the three regions of Abu Dhabi: the Abu Dhabi region, Al Ain region, and Al Dhafra region. The survey team made extra efforts to reach all community residents to achieve representative samples. The survey was available in Arabic, English, and six other Asian languages. The survey was distributed online. More than 50 survey links were created and distributed amongst the various segments of the community. Both DCD and SCAD were involved in distributing the survey links. DCD also sent encouraging calls to the communities, inviting their participation in the survey. Means of survey distribution included phone calls, messengers, emails, and social media. Survey representatives also appeared in several national TV newscasts to encourage participation. It should be added here that the online means of distribution also facilitated reaching respondents who were not in the country at the time of distribution. A total of 72,034 respondents participated in the survey.

### 3.2. Design and Analysis

The analytical approach adopted for this study hypothesizes that health and happiness are associated with other well-being factors of mental feelings and habits related to dietary food, with sport and exercising acting as significant determinants. Adopting a path model framework offers distinct advantages. First, it allows us to investigate patterns and directions of associations given the model considered. Second, it offers a different examination of the relationships between the variables. Third, it examines the impact of a set of predictor variables on multiple dependent variables.

The main hypotheses test the existence of direct relations between happiness and variables of physical activities, self-rated health, positive and negative mental feelings, and eating healthy food. In addition, the relations between self-perception of health and other variables will also be explored. We could summarize research objectives as:

- Design a path model of happiness and subjective health, and the multiple associations of mental health, exercising and eating healthy.
- Determine the outcome direction of association between happiness and health.
- Clarify the association strength of the established directions.
- Determine the direction of association between the factors of mental health, exercising, and eating healthy, with the two main factors of happiness and health.

Some pre-analysis attempts and examinations were carried out before initiating the path analysis. The pre-analysis included correlations and simple and multiple regression analyses. As the result of the pre-analysis, some variables were dropped from further path analysis. We need to elaborate that correlation coefficients measure the absolute value of the correlation between variables in a given body of data. However, a path coefficient measures the direct influence of one variable upon another and permits the correlation coefficient’s separation into direct and indirect effects components. It should be mentioned here that some of the pre-analysis regression results were strongly confirmed by the final path analysis. For example, and most specifically, regression analysis having happiness as the dependent variable recognized all seven determinants used in the path analysis framework as significant. The final proposed path model hypothesizes that happiness is associated with specific variables. The model was tested using LISERL v.10. [77]. The maximum likelihood estimation was employed because of its advantage of allowing for the simultaneous examination of both indirect and direct effect paths present throughout the model. This maximum likelihood estimation also allows us to test the overall fit of the data to the hypothesized model [78].

We used several fit indices to assess the overall path model fit. First, the chi-square statistic was used to evaluate the magnitude of discrepancy between the data sample used and the covariance matrix predicted by the model [79]. Second, as suggested by [80], the chi-square/degree of freedom ratio (CMIN/DF) was used to assess model fit further. The threshold of 3.0 was followed, as suggested by [78]. Other fit statistics included the Normed Fit Index (NFI), Non-Normed Fit Index (NNFI), Comparative Fit Index (CFI), Goodness of Fit Index (GFI), and the Adjusted Goodness of Fit Index (AGFI). To indicate a good fit, the minimum value for these indices should be greater than or equal to 0.95, and a minimum value of 0.90 indicates adequate fit [79,80]. The Root Mean Square Error of Approximation (RMSEA), which estimates the average absolute difference between estimated model covariances and the observed covariances, was also checked. A value less than 0.06 indicates a good model [80]. The same logic uses the Root Mean Square Residual (RMR). Finally, to test the null hypothesis, we considered a *p*-value testing the null hypothesis (PCLOSE) of the RMSEA. Many researchers recommend a non-significant result greater than 0.05 required to reject the null [77].

### 3.3. Variables in the Final Path Model

Based on the presented extensive review of the literature and the objective of this research, several variables were selected from the QoL survey. The two main variables are subjective self-rated health (1–5-point scale) and subjective happiness (0–10-point scale). The model also includes four subjective mental health variables (often feeling calm and peaceful, often having lots of energy, often feeling downhearted and depressed, and often feeling rushed or pressed for time), where each is evaluated on a (1–5-point scale). The other two variables include often eating healthy food and doing physical activity sport (minimum 30 min). Both are rated using (1–5-point scales). Since the scales for variables were different, the data were standardized for further path analysis. The descriptive characteristics of the variables in the model are presented in Table 1. The table shows descriptive statistics of the variables that remained in the final path analysis. As mentioned, all variables except the happiness variable used scales ranging from 1 to 5. Out of the eight variables in the path model, two mental feelings variables were worded negatively (how often feeling downhearted and depressed, and how often feeling rushed or pressed for time).

## 4. Results

Table 2 shows the breakdown regarding specific categories. About 62.1% were male, and 37.9% were female. Most of them were married (80.9%), while only 14.3% were single. Around (4.8%) were separated, widowed, or divorced. About 43.5% were Emirati, and 56.5% were non-Emiratis. Regarding education attainment, the most significant percentage (38.4%) of the elderlies held a bachelor’s degree, while 3.2% held doctorate degrees. Those not holding any degrees below bachelor’s degrees accounted for 48.1%. Most of the respondents (65.2%) were between 30 and 44 years old. About 73.6% of respondents resided in Abu Dhabi, 22% resided in Al Ain, and 4.4% resided in Al Dhafra. It should be realized that the percentages of the different categories reflect the accurate representations of each in Abu Dhabi. However, Emiratis in Abu Dhabi are less than the accurate representation reflected in the response rates. Therefore, specific weighing was used to represent the actual percentages for this category.

Table 2 also provides the happiness scores for each respondent category. Happiness scores favor females (7.308 relative to 7.009). The widowed and singles recorded the highest means (7.495 and 7.241, respectively), while the divorced and separated recorded means of 6.861 and 6.433, respectively. The marred respondents recorded happiness means closer to the medium point (7.138). Regarding education attainment, those with higher degrees, such as bachelor, master, and doctorate, recorded the lowest happiness (7.043, 6.944, and 6.937 relatively). On the other hand, those with school degrees enjoyed the highest mean of (7.629). The oldest category of respondents enjoys the highest happiness scores along with school-aged children (7.753 and 7.785, respectively). Meanwhile, non-Emiratis recorded higher means than Emiratis (7.324 relative to 7.008). Finally, the figures show that those residing in Abu Dhabi city recorded the lowest happiness means relative to those in Al Ain or Gharbia (7.031, 7.194, and 7.186, respectively). 

The path analysis used the covariance matrix shown in Table 3. A covariance value reflects the relationship of two variables whenever one variable changes. When an increase in one variable results in an increase in the other variable, both variables have positive covariance.

The final path model fit statistics are shown in Table 4. The fit statistics reflect an excellent model in all aspects. The RMSEA is 0.00413. The value of CMIN/DF is 1.537, which is far below the threshold of 3.0. All fit statistics are above 0.99, which indicates an excellent model. It should be noted that since many studies identified the direction of association to be from happiness to health, we also tried to explore that specific direction. The analysis provided a Root Mean Square Error of Approximation (RMSEA) of 0.0938, with many of the fit indices between (0.806) and (0.884). 

Table 5 shows the standardized path statistics and their related t-values. Overall, all paths point to happiness and self-rated health. A total of five variables point to happiness, while six points to self-rated health. In general, it is interesting to note the presence of all four mental-health-related variables in the final path model and their significant association with both happiness and self-rated health. The most significant total association with either happiness or health is the mental feeling of how often one feels they have lots of energy. The other mental sense of how often one feels downhearted and depressed presented itself with happiness’s most significant negative association.

Concerning happiness, the largest direct association is from “how often feeling downhearted and depressed” with a pessimistic standardized estimate of −0.1707. The other significant estimates are from “how often feeling having lots of energy”, “how often feeling calm and peaceful”, “how often doing physical activity/sport”, and “how often eating healthy diet/food”, with estimates of 0.1392, 0.1314, 0.0759, and 0.0385, respectively. In summary, all four mental-feeling-related variables have a significant presence in the happiness of Abu Dhabi respondents. However, it is essential to see that “self-rated health” is also associated with happiness. As a result, we need to calculate the indirect association of these variables with happiness but through self-rated health. Table 4 shows the indirect and total associations of these variables with happiness. Five variables have both direct and indirect associations with happiness. The indirect associations are through the mediation of the self-rated health variable. The highest indirect association is from “how often feeling having lots of energy” (0.0293). The others are from “how often feeling calm and peaceful” (0.0194), “how often eating healthy diet/food” (0.0153), “how often doing physical activity/sport” (0.0115), and “how often feeling downhearted and depressed” (−0.0107). 

In path analysis, a path coefficient indicates the direct effect (or association) on another variable. Path coefficients are standardized because they are estimated from correlations (a path regression coefficient is unstandardized). Path coefficients are written with two subscripts. A total of six variables contribute to the respondents’ self-rated health indicator. The highest contribution is from “how often feeling having lots of energy”, with a standardized statistic of 0.1944. The other five significant estimates are related to “how often feeling calm and peaceful”, “how often eating healthy diet/food”, “how often feeling downhearted and depressed”, “how often doing physical activity/sport”, and “how often feeling rushed or pressed for time”, with standardized estimates of 0.1485, 0.1011, −0.0715, 0.0467, and −0.0239, respectively. 

**Figure 1:**
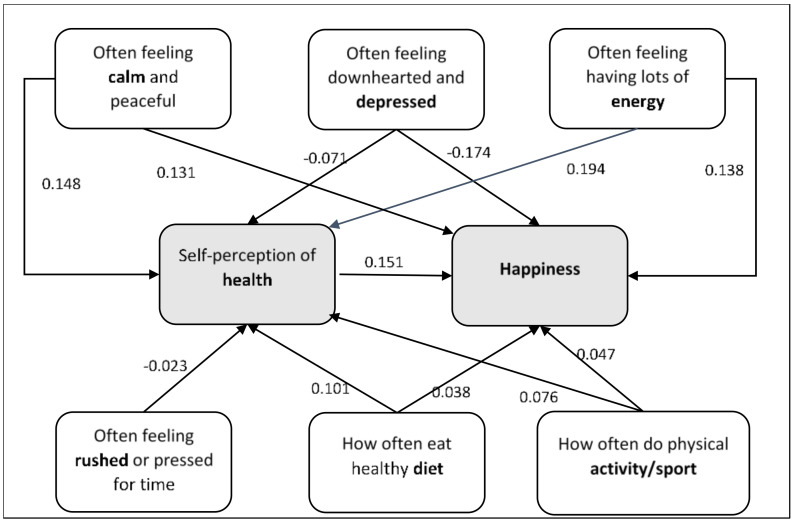
The final path model.

Figure 1 shows that the four mental feeling variables act independently with no paths relating directly to each other. There are also no associations between these mental health variables and “how often eating healthy food/diet” and “how often doing physical activity/sport” directly or indirectly. We should also note that the mental feeling variables directly correlate with happiness and self-perception of health, except that “how often feeling rushed or pressed for time” has no direct association with happiness.

## 5. Discussions

The most important objectives of this study were to explore how feelings of happiness are associated with other factors of self-rated health, subjective mental feelings, being active and practicing sport, and consuming healthy diets. In addition, the aim was to extend our knowledge about the associations between and further exploit the directionality of the relationships, especially between happiness and self-rated health. We have demonstrated that self-rated health and happiness are related to one another. The results indicate a direct association between most of the variables and happiness. In addition, we also demonstrated that self-perception of health mediates between happiness and other variables of physical activity, eating healthy, and mental feelings. The path model, featured with six variables interacting with self-rated health and happiness, shows excellent fit statistics of the path analytic model. 

The findings show that self-rated health influences happiness with many other healthy lifestyle practices and patterns. In other words, self-rated health might explain happiness to a large extent. The significant association between self-perception of health and happiness is no surprise, as it is consistent with the results from other studies [39]. Thus, the study offers further support to the call by many researchers that social policymakers should consider the relationship between self-rated health and happiness in their policy interventions [41]. As reviewed earlier, many studies pointed to the direction of association to be from happiness to self-rated health. Our further sensitivity analysis for exploring that direction provided a path model that was less significant than the one proposed here. Again, this might raise a debate that this specific direction might also depend on the presence and interaction between the other variables present in the model (subjective happiness, self-rated health, four types of mental health variables, eating healthy food, and practicing sports). 

The results also align with relevant findings that explain the association of happiness with health and positive emotions [49,50]. Several variables of mental feelings have both direct and indirect associations with happiness. Such outcomes agree with the concept that mental health or feelings are subjective [48]. The results support other findings that attribute happiness as significant reflections and aspects of positive psychology and human emotions [49,50]. 

The direct association between mental feelings and self-rated health is evident in this Abu Dhabi research, which is congruent with other international applications that confirm that those general mental feelings are a reliable subjective indicator of self-rated health [81].

The present study results also reflect the significance of eating healthy food on both self-rated health and happiness. Such outcomes go parallel with other studies that note the association between positive well-being and healthy food consumption [61] and the literature confirming the association of consuming diet-rich food with a range of health benefits [63,65] and happiness [8].

The resulting general associations between physical activity and happiness align with many studies that have observed positive associations between self-reported physical activity and happiness [55,82]. The findings could encourage policymakers to offer innovative solutions for different categories of citizens, believing that a physically active lifestyle contributes to increased happiness. However, the results do not provide paths from physical activity to the four mental feelings variables, which is not consistent with a large body of literature demonstrating that physical activity effectively reduces depression and anxiety [59,60]. We should stress, however, that the path analysis in several other similar studies also does not confirm the causal relationship between physical activity and happiness [58]. In addition, our path model did not reveal any association between the four mental feelings variables, either directly or indirectly, and eating healthy food and physical activity. It is worth noticing that the mental feeling variables have direct associations with both happiness and self-perception of health, except that “often feeling rushed” shows no direct association with happiness. 

The most apparent outcome of this research is the significance of the associations between all seven variables in the model with both happiness and self-rated health. In addition, many variables show an indirect association with happiness. The containment of all variables to show associations to happiness directly or indirectly provides much support for the Better Life framework hypothesized by the OECD. Such support is also declared by other related research in other countries [14,15]. This supporting evidence provides much-needed influence and capacity to social policymakers to convince community members of the value of their strategies associated with advancing happiness in their community. 

In general, finding the correct meaning of the results of this research is undoubtedly a challenge for social policymakers. Social policymakers would utilize the results effectively by looking at possible ways to further invest in happiness-related health policies. The results reflect the belief that happy people are more likely to feel healthier, show positive mental feelings, live a healthy lifestyle by exercising correctly and avoid eating unhealthy food. Policymakers need to understand the interrelations between these factors when designing their public awareness programs toward a more positive lifestyle.

The findings of this research concerning the association of happiness to health, mental health, sport and exercise, and eating healthy could encourage investing in related social policies to guarantee the cohesive interaction between related decision-making bodies to play their role in the well-being of people.

The lack of other studies focusing specifically on happiness and self-rated health analyzed here in the Abu Dhabi respondents constitutes a limitation of the discussion of the current study’s findings. This is especially true for self-rated health status. Some might argue that it might lack greater clarification, primarily used in parallel with physical activities and mental health factors. Nevertheless, the results point to the need to further explore the direction of relations between happiness and self-rated health. Expanding the path analysis model with relevant indicators could add information to policymakers to better understand the direction of associations between the relevant well-being factors.

The results addressed differences in happiness between the various categories of gender, age, marital status, education attainment, place of residence, and nationality (Emirati or non-Emirati). The current research did not address these facts, as more specific objectives were addressed by using path analysis. Future research could look at these differences with more methodological designs to better understand their various well-being determinants, including happiness. In addition, an expanded path model could be designed to consider all the different respondent categories. 

Future research should focus on analyzing the differences when considering happiness, self-rated health, mental feelings, and supportive actions for the different categories of people living in Abu Dhabi. The categories could include gender, age, marital status, ethnicity, education attainment, living region, type of housing, income class, specific life habits, and other factors. As a result, future research could initiate such studies on older adults or younger school-aged children. Such in-depth studies might enhance and prolong the value of awareness campaigns associated with social policies. Moreover, the study used single-item indicators of happiness, self-rated health, mental feelings, and the habits of eating healthy and exercising. Future research could try multi-item scales to cover more profound perceptions and understandings. Finally, future research could refer to more longitudinal studies to detect developments or progress in the target population’s characteristics in Abu Dhabi. Such studies that extend beyond a single moment in time might reflect more accountability and meanings to social policy outcomes. It is also worth mentioning that the COVID-19 pandemic has played a significant role in the happiness and life satisfaction of Abu Dhabi residents (reference). We also recommend an extension (or trended) version of this research to elaborate on the significance of the COVID-19 pandemic regarding all eight well-being determinants used in this study. 

## 6. Conclusions

Consistent with many other studies, the results suggest that multiple factors explain happiness. Through path analysis, this study demonstrates the relations between happiness, physical activities, eating healthy, and certain mental feelings. Several significant direct or indirect paths observed in other international studies were not observed in our path analysis. As a result, this research could partially support the findings observed elsewhere. Moreover, the resulting path model’s overall excellent fit may provide sound understandings leading to constructive policies. A better understanding of the associations presented in this study can help policymakers develop more effective health and social intervention programs for different segments of the community.

With recognized relationships between happiness, health, and physical activities, policymakers in the different social sectors are encouraged to envision and design more innovative physical activities and healthy eating behaviors to enhance happiness and health perception. In particular, in the education sector, students should be provided with various opportunities to become more active at schools and universities. Baselines should be established to monitor changes in physical activity patterns over time. 

We should note that the current study is amongst the first to address happiness in a culture such as the UAE. According to several authors, happiness and quality of life are significantly culturally rooted (Ye et al., 2014). Nonetheless, this study contributes to the literature for the first empirical analysis of the relationship between happiness and self-rated health in Abu Dhabi. The study also provided significant insights into subjective mental health roles on happiness and self-rated health. In addition to practical implications, the present study also contributed to the existing literature, as it enhances our current understanding of the interlink between the various well-being constructs. The holistic path analysis of this study added to existing research by identifying a group of eight significant constructs.

The roles of other related factors, such as eating healthy and exercising, were also considered to be significant. Further research could address the influential roles of some categorical differences that might affect happiness and self-rated health, such as gender, age, marital status, and income. Such a review might focus on the results for issuing more effective social policies. Despite this reasonable attempt, relationships between happiness, physical activities, mental well-being, and self-perception of health in Abu Dhabi have remained largely unexplored. Moreover, the current research has limitations when it tested one single path model for the whole community in Abu Dhabi without further consideration of the specific categories or segments of the community. Therefore, future research should further explore such path models for each of the different categories of people in the community, which may differ by social status, education level, place of living, income, work, and housing type. In addition, and to support exceptional policies and strategies, more research on the association between physical activity and happiness and health should be conducted. In other words, promoting sports and well-being might require more rigorous efforts from social policymakers for long-term strategies. As a final remark, it is generally accepted that happiness is perceived differently in individualistic, secular countries (West) and collectivist ones with a significant influence of religion on people’s lives (East and Southeast Asia). Since the QoL survey could be analyzed according to this crucial factor, future research could investigate if cultural differences could influence happiness and its related determinants.

## Figures and Tables

**Table 1:** Descriptive statistics of variables in the path model.

	Symbol	Scale	Mean	S.D.
Self-perception of health	HLTH	1–5	3.3198	1.0635
Happiness	HPNS	0–10	7.1437	2.5265
How often feeling calm and peaceful	CALM	1–5	3.0680	1.0346
How often feeling having lots of energy	ENRG	1–5	3.2076	1.0053
How often feeling downhearted and depressed	DPRS	1–5	2.8072	1.0556
How often feeling rushed or pressed for time	RUSH	1–5	3.2472	1.0784
How often eating healthy diet/food	FOOD	1–5	3.4497	0.9992
How often doing physical activity/sport (minimum 30 min)	SPRT	1–5	2.6101	1.3452

**Table 2:** Respondent’s profile.

Gender	Percentage	Happiness Score
Male	62.1%	7.009
Female	37.9%	7.308
Marital status		
Married	80.9%	7.138
Single	14.3%	7.241
Divorced	3.7%	6.861
Separated	0.6%	6.433
Widowed	0.5%	7.495
Education level		
Illiterate	1.7%	7.473
Below secondary school	5.2%	7.629
Secondary school	14.6%	7.345
Post high school training certificate	16.7%	7.179
College diploma	9.9%	7.035
Bachelor’s degree	38.4%	7.043
Master’s degree	9.3%	6.944
Doctorate degree	3.2%	6.937
Age		
15–19	0.20%	7.785
20–24	2.1%	7.384
25–29	9.7%	6.993
30–34	20.0%	6.861
35–39	24.9%	6.999
40–44	20.3%	7.129
45–49	12.2%	7.277
50–54	6.4%	7.513
55–59	2.7%	7.639
60+	1.3%	7.753
Nationality		
Emirati	43.5%	7.008
Non-Emirati	56.5%	7.324
Living region		
Abu Dhabi	73.6%	7.031
Al Ain	22.0%	7.194
Al Dhafra	4.4%	7.186

**Table 3:** The covariance matrix.

	HLTH	HPNS	CALM	ENRG	DPRS	RUSH	FOOD	SPRT
HLTH	0.993							
HPNS	0.274	0.990						
CALM	0.306	0.300	0.927					
ENRG	0.333	0.299	0.512	0.948				
DPRS	−0.241	−0.308	−0.389	−0.357	0.998			
RUSH	−0.168	−0.169	−0.311	−0.224	0.433	0.943		
FOOD	0.210	0.147	0.204	0.244	−0.200	−0.151	0.928	
SPRT	0.194	0.082	0.188	0.252	−0.176	−0.144	0.280	0.962

**Table 4:** Path model goodness-of-fit statistics.

Fit-Statistics	Value
Degrees of Freedom	1
Maximum Likelihood Ratio Chi-Square (CMIN/DF)	1.537
The *p*-value	0.2151
Root Mean Square Error of Approximation (RMSEA)	0.00413
Normed Fit Index (NFI)	0.998
Non-Normed Fit Index (NNFI)	0.999
Comparative Fit Index (CFI)	0.996
Goodness of Fit Index (GFI)	0.994
Adjusted Goodness of Fit Index (AGFI)	0.998
Root Mean Square Residual (RMR)	0.000897

**Table 5:** Model fits and statistics.

From	To	Direct Association	t-Value	Indirect Association	Total Association
How often feeling calm and peaceful	Happiness	0.1314	19.740	0.0194	0.1508
How often feeling calm and peaceful	Health	0.1485	22.090	-	0.1485
How often feeling having lots of energy	Happiness	0.1392	19.446	0.0293	0.1685
How often feeling having lots of energy	Health	0.1944	29.359	-	0.1944
How often feeling downhearted and depressed	Health	−0.0715	−11.556	-	−0.0715
How often feeling downhearted and depressed	Happiness	−0.1747	−30.584	−0.0107	−0.1854
How often feeling rushed or pressed for time	Health	−0.0239	−3.787	-	−0.0239
How often eating healthy diet/food	Health	0.1011	17.622	-	0.1011
How often eating healthy diet/food	Happiness	0.0385	6.633	0.0153	0.0538
How often doing physical activity/sport	Health	0.0467	8.368	-	0.0467
How often doing physical activity/sport	Happiness	0.0759	13.524	0.0115	0.0874

## Data Availability

The data presented in this study are available on request from the corresponding author. The data are not publicly available due to privacy restrictions.
